# Longitudinal trends in antibiotic resistance of *Escherichia coli*: A 10-years retrospective analysis in a tertiary care hospital in Lahore, Pakistan

**DOI:** 10.12669/pjms.41.11.12000

**Published:** 2025-11

**Authors:** Alishba Fayyaz, Mariam Danish Iqbal, Farkhanda Ghafoor

**Affiliations:** 1 Alishba Fayyaz, MS Department of Research and Innovation, Shalamar Medical and Dental College, Lahore, Pakistan; 2 Mariam Danish Iqbal, MBBS Microbiology Diagnostic Laboratory, Shalamar Medical and Dental College, Lahore, Pakistan; 3 Farkhanda Ghafoor, PHD Department of Research and Innovation, Shalamar Medical and Dental College, Lahore, Pakistan

**Keywords:** Antibiotic resistance, *Escherichia coli*, Retrospective analysis

## Abstract

**Objective:**

In Pakistan, the rising rate of *Escherichia coli* (*E. coli*) resistance has been reported, leading to limited treatment options and contributing to a high morbidity and mortality ratio. This study aimed to analyze changes in antibiotic resistance patterns against commonly prescribed antibiotics in a tertiary care hospital in Lahore, Pakistan, over the span of 10 years.

**Methodology:**

A retrospective cross-sectional study was designed to analyze a decade-long (2013-2022) resistance trend of *E. coli* isolates identified at a tertiary care Shalamar Hospital in Lahore, Pakistan. The frequency of *E. coli* isolated from different infectious sites across various age groups and their resistance proportions against CLSI-recommended antibiotics was accessed for 10 years.

**Results:**

*E. coli* is a Gram-negative bacterium that has caused a wide range of infections, predominantly urinary tract infections, with increasing antibiotic resistance. The results indicate a resistance rate of 98% to ampicillin, 93% to amoxicillin/clavulanic acid, and 87% and 82% to second and third-generation cephalosporins: cefuroxime, cefotaxime and ceftriaxone, respectively. High susceptibility against carbapenems and polymyxins drugs was also reported. However, over 10 years, an increasing resistance trend was observed against cefuroxime and imipenem and a significant decreasing trend was observed against gentamicin and nitrofurantoin. Likewise, resistance against beta-lactam like cefotaxime, ceftriaxone, and cefuroxime significantly increased compared to imipenem and meropenem compared to mean antibiotic resistance across all age groups.

**Conclusion:**

It highlights the emerging resistance trend against commonly prescribed antimicrobial drugs, leading to understanding the longitudinal resistance trends of *E. coli* in the region.

## INTRODUCTION

Antibiotic resistance is one of the major threats to humanity, especially in underdeveloped countries. The plausible causes of antimicrobial drug resistance are unnecessary usage and easy availability of antibiotics in local pharmacies, increasing global mobility, neglected sanitation and secretion of un-metabolized antibiotic residuals in the environment.^1^,^2^ *Escherichia coli* (*E. coli*) is an important commensal in the gut microbiome and an infectious Gram-negative pathogen that can cause wide range of infections in humans like urinary tract infections (UTIs), intestinal diseases and bloodstream infections. Antibiotic resistance against *E. coli* has been causing an alarming situation in managing the infection by limiting the treatment choices.^3^ *E*. *coli* harbor various mechanisms to overcome being destroyed by antibiotics, like mutating the binding site in the case of quinolones or by producing beta-lactamase and extended-spectrum beta-lactamase enzymes that can hydrolyze beta-lactam and broad-spectrum beta-lactam drugs. *E. coli* strains that produce extended spectrum beta lactamases pose significant treatment challenges as these strains are resistant to multiple antibiotics and have virulence factors contributing towards critical infections.^4^ Strains of *E. coli*, which produce the emergence of extended-spectrum β-lactamase (ESBL), are also strongly linked to resistance against aminoglycosides, sulfonamides and fluoroquinolones.^5^

According to the Centers for Disease Control and Prevention (CDC), approximately 20-50% of all recommended antibiotics to critical care patients in United States hospitals are either unnecessary or unsuitable.^6^ If such challenges exist in developed countries, they are likely to be far more pronounced in under developing nations like Pakistan , which lack the public awareness, infrastructure, and resources needed to manage antibiotic resistance effectively. Therefore, the objective of this study was to understand the burden of *E.coli* infections on the local healthcare system and to identify the change in antibiotic resistance pattern of Clinical Laboratory & Standards Institute (CLSI) guided panel of antibiotics against *Escherichia coli* from 2013 to 2022 for a better management plan to tackle *E.coli* infections and its associated complications.

## METHODOLOGY

A retrospective analysis was conducted on the data derived from Electronic medical records from Shalamar Hospital in Lahore, Pakistan.

### Data collection:

*E. coli* isolates were collected from various clinical specimens including urine, blood, wound swabs, pus and sputum. The *E. coli* antibiotic susceptibility data for the CLSI-recommended panel of antibiotics from 2013 to 2022 were included, comprising amikacin, amoxicillin/clavulanic acid, ampicillin, cefotaxime, ceftriaxone, cefuroxime, ciprofloxacin, colistin, co-trimoxazole, gentamicin, imipenem, meropenem, nitrofurantoin, and piperacillin/tazobactam.^7^

### Ethical approval:

A prior ethical approval Reference number, SMDC-IRB/AL/17/2023, dated July 6, 2023 has been granted by the Institutional Review Board, Shalamar Medical and Dental College.

### Microbiological methods:

*Escherichia coli* was identified using a combination of standard biochemical tests and the VITEK 2 automated system (bioMérieux). Routine biochemical tests included the IMViC series (indole, methyl red, Voges-Proskauer, and citrate utilization), lactose fermentation, triple sugar iron (TSI) agar test, and urease test to confirm *E. coli* identity. The VITEK 2 system, using GN identification cards, was employed for automated species confirmation based on biochemical profiles. Antibiotic susceptibility testing (AST) was performed using the VITEK 2 system with AST-GN cards, following Clinical and Laboratory Standards Institute (CLSI) guidelines to determine resistance and sensitivity profiles for the recommended panel of antibiotics.^7^

### Inclusion criteria:

Calculating the proportion of antibiotic resistance depends on two prerequisites: consistent data is available for every year from 2013 to 2022, and at least 30 isolates are identified per year for each antibiotic.

### Statistical analysis:

The data was retrieved and managed in Microsoft Excel 2016. Percentage of resistant and sensitive *E. coli* Isolates identified from year 2013 to 2022. Analysis of variance and R^2^ value was computed to identify significant changes in linear antibiotic resistance trends. T-test was used to determine beta-lactam antibiotics resistance compared to mean antibiotic resistance of isolates by age of patients categorized by decades of life. Graphs were generated using Microsoft Excel 2016.

## RESULTS

### Demographics of Data:

**Fig.1:**
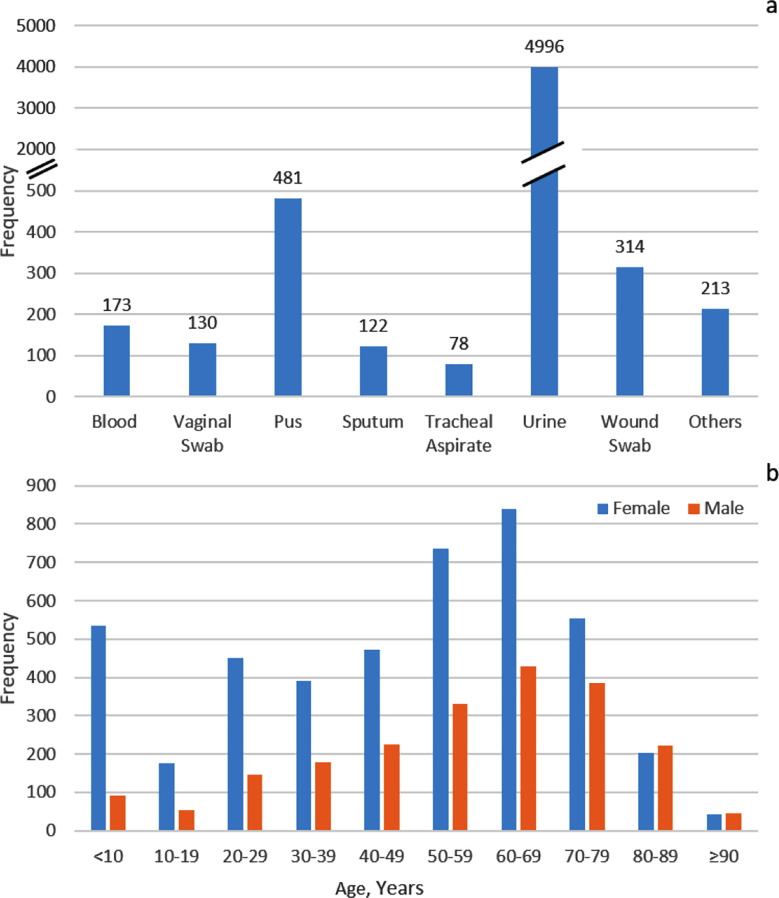
(a) Distribution of Escherichia coli isolates across different clinical specimen types. (b) Distribution of Escherichia coli isolates stratified by patient age groups and gender, showing the relative burden of infections across demographics.

A total of 6507 *Escherichia coli* isolates were recorded from 11 different patient sites, visiting the Shalamar hospital diagnostic laboratory (Fig.1a). Among these sites, 4996 (76.8%) *E. coli* isolates were identified in urine samples. Patient age was known for all isolates, with 626 isolated from patients younger than 10 years, and 1269 E. coli isolates were obtained from patients belonging to the age group 60-69 years. Of 6507 patients from whom isolates were obtained, 4397 (67.6%) were females, and 2110 (32.4%) were males (Fig.1b).

### E. coli Antibiotic Resistance Rates:

**Fig.2:**
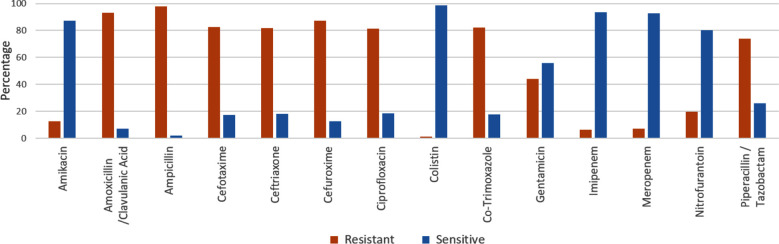
Resistance profile of *Escherichia coli* isolates showing the percentage of resistance to different antibiotic.

Within the recorded study data from January 2013 to December 2022, the percentage of resistant and sensitive isolates to clinically relevant antibiotics are shown in Fig.2. Among tested *E. coli* isolates, 98% were resistant to ampicillin, and 93% were resistant to amoxicillin/clavulanic acid. Increased resistance rate was also observed against second-generation cephalosporin: cefuroxime (87%) and third-generation cephalosporin: cefotaxime and ceftriaxone (82%). However, last-resort antibiotics like imipenem, meropenem and colistin have shown negligible resistance rates of 6%, 7% and 1%, respectively.

### Trend in resistance:

**Fig.3:**
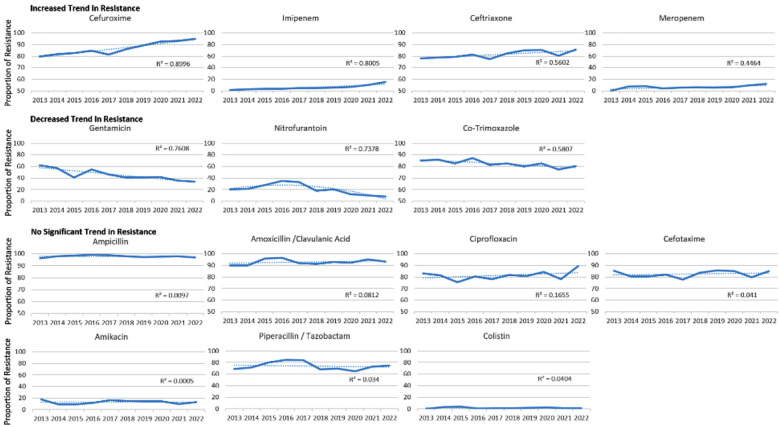
Antibiotic Resistance Among Escherichia coli Isolates from 2013 to 2022. p values were calculated using analysis of variance to identify significant linear trends in antibiotic resistance.

The antibiotic resistance rates by years (Fig.3, Table-I) show, *E. coli* resistance against cefuroxime has increased from 80% to 95% between 2013 to 2022 (R^2^ = 0.8996, *p* = <0.001). Imipenem resistance increased from 1% to 15% (R^2^ = 0.8005, *p* = <0.001). Meropenem resistance increased to 12% in 10 years (R^2^ = 0.4464, *p* = 0.029). However, from the year 2013 to 2022, decreasing resistance trend was observed against gentamicin (62% to 34%, R^2^ = 0.7608, *p* = 0.001), nitrofurantoin (20% to 8%, R^2^ = 0.7374, *p* = 0.047) and co-trimoxazole (85% to 80%, R^2^ = 0.5807, *p* = 0.011). No significant changes in resistance was observed against ampicillin, amoxicillin/clavulanic acid, ciprofloxacin, cefotaxime, amikacin, piperacillin/tazobactam and colistin.

**Table-I:** Antibiotic Resistance Percentage Among *Escherichia coli* Isolates from 2013 to 2022.

Years	Amikacin	Amoxicillin/ Clavulanic Acid	Ampicillin	Cefotaxime	Ceftriaxone	Cefuroxime	Ciprofloxacin	Colistin	Co-Trimoxazole	Gentamicin	Imipenem	Meropenem	Nitrofurantoin	Piperacillin/ Tazobactam
2013	17	90	96	85	78	80	83	0	85	62	1	0	20	69
2014	9	90	98	80	79	82	81	3	86	57	3	7	21	71
2015	9	96	98	80	79	83	75	3	83	41	4	8	28	80
2016	11	96	99	82	81	85	80	1	87	55	3	4	35	84
2017	17	92	99	78	78	81	78	1	81	46	5	5	33	84
2018	15	91	98	84	82	86	82	1	83	41	5	6	18	68
2019	14	93	97	86	85	89	81	2	80	41	6	5	21	70
2020	14	92	98	85	85	92	84	2	83	41	7	6	12	65
2021	10	95	98	80	80	93	78	1	77	36	10	10	10	73
2022	13	93	97	85	85	95	89	1	80	34	15	12	8	75

### Antibiotic Resistance Rate by Patient Age:

**Fig.4:**
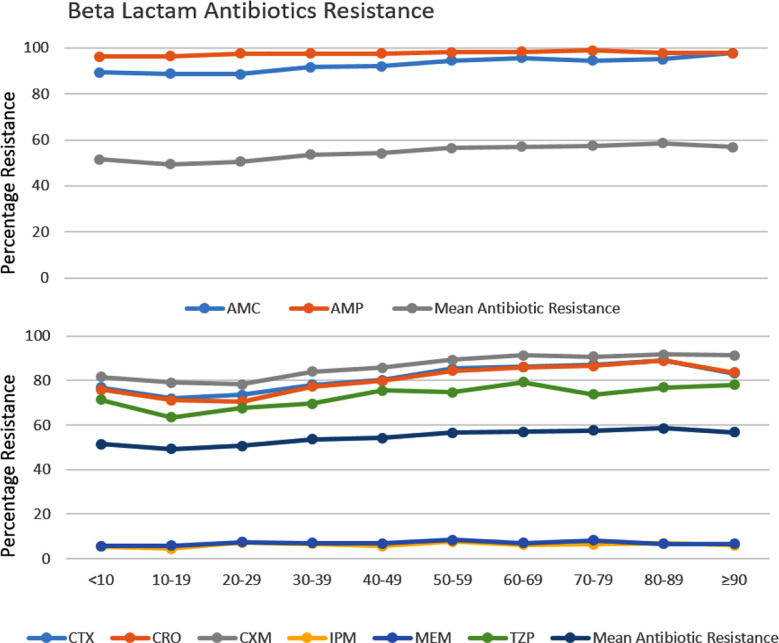
Mean resistance of the beta-lactam class versus the resistance of each individual beta-lactam antibiotic across age groups among *Escherichia coli* isolates. p values were calculated using a T-test to identify significant differences between mean beta lactams antibiotic resistance and individual antibiotic resistance (all p = <0.001). AMC: Ampicillin, AMP: Amoxicillin/Clavulanic Acid, CTX: Cefotaxime, CRO: Ceftriaxone, CXM: Cefuroxime, IPM: Imipenem, MEM: Meropenem, TZP: Piperacillin/Tazobactam.

Beta-lactam antibiotics like ampicillin and amoxicillin/clavulanic acid reflect significantly higher resistance rates in comparison to mean antibiotic resistance (Fig.4, Table-II). Similarly, cefotaxime, ceftriaxone, cefuroxime and piperacillin/tazobactam has also shown significantly increased resistance than imipenem and meropenem in comparison to mean antibiotic resistance across all age groups (Fig.4).

**Table-II:** Percentage resistance of beta-lactam antibiotic across age groups among *Escherichia coli* isolates.

Age Groups	Ampicillin	Amoxicillin/ Clavulanic Acid	Cefotaxime	Ceftriaxone	Cefuroxime	Imipenem	Meropenem	Piperacillin/ Tazobactam	Mean Beta Lactam Resistance
<10	89	96	77	76	82	5	6	71	51
10-19	89	96	72	71	79	5	6	64	49
20-29	89	97	73	71	78	7	7	68	51
30-39	92	97	78	77	84	7	7	70	54
40-49	92	97	80	80	86	6	7	75	54
50-59	94	98	85	84	89	8	8	75	57
60-69	96	98	86	86	91	6	7	79	57
70-79	94	99	87	87	91	7	8	74	58
80-89	95	98	89	89	92	7	7	77	59
≥90	98	98	83	84	91	6	7	78	57

## DISCUSSION

Increasing antimicrobial resistance is a public health emergency. Excessive and inappropriate usage of antibiotics resulted in a continuous increase of antimicrobial resistance that has led to the evolution of multidrug-resistant *E. coli* strains. As a result of alarmingly high resistance, infections that were once manageable have now become either incurable or require last-line antibiotics, raising serious concerns for future treatment options.^8^ *E. coli* is a Gram-negative bacillus that can cause many extra-intestinal infections across all ages. According to WHO, enhanced resistance against *E. coli* is gaining momentum as a critical global threat because of international reports of multidrug, extreme drug and pan-drug resistant isolates of *E. coli*.^9^-^11^

*E. coli* is one of the major causative agents of urinary tract infections that affect people; therefore, UTIs are 2nd leading reason for antibiotic prescription, and females are more susceptible to UTIs because of their anatomical difference and shorter urethra.^12^-^14^ Our data showed that the highest number of *E. coli* isolates were identified in urine samples, with a higher prevalence in female patients (Fig.1).

Antibiotics such as cefotaxime, ceftriaxone, and cefuroxime, are among the most commonly prescribed agents for treating infections caused by Gram-negative bacteria, particularly *E. coli*. However, *E. coli* can develop resistance to these drugs by producing β-lactamases. 15 The standard recommendations specify that an antibiotic must have >90% sensitivity towards infectious *E. coli* in that population to be used as an empirical antibiotic.^16^

Our data indicates the high resistance rate against commonly prescribed antibiotics like amoxicillin/clavulanic acid (93%), ampicillin (98%), cefotaxime (82%), ceftriaxone (82%), cefuroxime (87%), ciprofloxacin (82%), co-trimoxazole (82%), piperacillin/tazobactam (74%) (Fig.2) suggesting to acquire alternate approach for treatment of *E. coli* infections. It is alarming that the resistance rate is significantly higher than that of other studies, which have indicated 19.4% and 11.7% resistance against amoxicillin/clavulanic acid and piperacillin/tazobactam, respectively.^17^ A research on urinary tract infections published from Karachi, Pakistan, highlighted that approximately 49% of Gram-positive and 57% of Gram-negative pathogens were resistant to ciprofloxacin.^13^ Quinolones, especially ciprofloxacin, is one of the most frequently prescribed first line of drugs to treat UTIs. Unfortunately, according to the 2017–2018 Global Antimicrobial Resistance and Use Surveillance System report, more than 70% of Pakistani *E. coli* strains were found to be ciprofloxacin-resistant.^13^ Another study suggested the gradual decrease in sensitivity of fluoroquinolones from 60% in 2005–2010 to 25% in 2017.^12^

According to a survey (2014-2018) use of antibiotics like cephalosporin and ciprofloxacin have increased by 1.86 defined daily dose units per 1000 individuals per day.^18^ National Action Plan Pakistan team also critically analyzed a situation in April 2018 and pointed out major factors responsible for enhanced antibiotic resistance, including inappropriate prescription, misleading marketing, and absurd usage of antibiotics in livestock and agriculture.^19^ To tackle antibiotic resistance in Pakistan’s infrastructure, collaborative working of all three key members of the healthcare facility: the clinicians, diagnosticians and pharmacists, is needed.^20^

There is slight hope when we analyze the efficacy of carbapenem drugs like imipenem and meropenem and polymyxin drugs like colistin that are still effective against ESBL (Fig.2 and Fig.4). Recent global reports are indicating a gradual increase against these drugs. According to one study, 9.4% of *E. coli* strains identified as ESBL were 50% and 75% resistant to imipenem and meropenem, respectively, thus making it very complicated to treat urinary tract infections.^21^ The results of this study are also worrisome, as resistance against meropenem over 10 years has drastically increased from 0% in 2013 to 12% in 2022. Imipenem has shown a similar trend with a resistance rate of 1% to 15% in 10 years. Compared to other beta-lactam medications, resistance to carbapenems is still manageable when treating *E. coli* infections, particularly urinary tract infections, provided a methodical approach is carried out (Figs. 3 and 4).

Javaid et al. detected the declining resistance trends to amikacin and gentamicin against *E. coli*, compared to other antibiotics with increased resistance. These results align well with our decreasing trend of gentamicin resistance, which has decreased from 62% to 34% in 10 years.^22^ While antimicrobial resistance is increasing for other major classes of drugs, the unexpected decreasing resistance trend against gentamicin, nitrofurantoin and co-trimoxazole in *E. coli* (Fig.3, Table 1), has been observed in this study, suggesting that continuous surveillance is needed to keep track of emerging drug resistance trends in *E. coli*. This may be due to decreased use of these specific antibiotics within the given time.

### Strengths and Limitation:

Our study addresses the long-term update on antibiotic resistance in a localized region in Pakistan that can help clinicians tailor their treatment options according to the regional resistance patterns. In addition to that, our study also highlights the consistently high resistance against commonly prescribed antibiotics like ampicillin amoxicillin, indicating the lack of real-time information availability to physicians to make informed decisions for patient health management. However, our research includes its retrospective nature and lack of in-depth analysis of *E. coli* urinary tract infections, especially in females. Nonetheless, this study provides a foundation for designing prospective, multi-centre studies and conducting molecular resistance profiling to understand resistance mechanisms better. It also emphasized the necessity for policymakers to establish and implement infection control procedures and antibiotic stewardship across the country to manage the growing threat of antimicrobial resistance to public health. It is also crucial to educate the community about the risks associated with self-medication and the excessive use of antibiotics.

## CONCLUSION

This study determined the *E. coli* resistance trend against beta-lactams, carbapenems, and aminoglycosides. Among these drugs, *E. coli* was extremely susceptible to Imipenem, Meropenem, and Colistin; however, a very high resistance rate was observed against Penicillin and Cephalosporins. This study has its strengths and limitations as it addresses the antibiotic susceptibility trends in ten years with a salient observation of a rapid increase in carbapenem resistance. However, large-scale research must be conducted to determine exact drug resistance patterns against *Escherichia coli* in Pakistan.

### Author’s Contribution:

**AF:** Conceived, designed, did statistical analysis & editing and writing of manuscript, is responsible for integrity of research.

**MD:** Did data collection. Literature search, and review the manuscript.

**FG:** Critical Review, analysis and final approval of manuscript.

All authors have read and approved the final manuscript and are responsible for the integrity of the study.
